# Cross-cultural Adaptation and Validation of the Malay Version of the Early Childhood Educators Child Abuse Questionnaire in Malaysia

**DOI:** 10.21315/mjms-12-2024-1009

**Published:** 2025-04-30

**Authors:** Siti Mariam Ja’afar, Azriani Ab Rahman, Wan Nor Arifin

**Affiliations:** 1 Department of Community Medicine, School of Medical Sciences, Universiti Sains Malaysia, Health Campus, Kelantan, Malaysia; 2 Biostatistics and Research Methodology Unit, School of Medical Sciences, Universiti Sains Malaysia, Health Campus, Kelantan, Malaysia

**Keywords:** child abuse, cross-cultural adaptation, early childhood, ECECAQ, validity, reliability

## Abstract

**Background:**

There is an increasing global trend of child abuse, and research into knowledge, attitudes, and intentions towards reporting child abuse among early childcare providers (ECPs) in Malaysia has been limited. There is no existing questionnaire in the Malay language that measures these aspects. Therefore, this study cross-culturally adapted and validated the Early Childhood Educators Child Abuse Questionnaire (ECECAQ) from English into Malay to assess knowledge and attitude towards child abuse and its reporting, as well as intentions to report child abuse among ECPs.

**Methods:**

This study comprises two phases. The first phase involved the cross-cultural adaptation of the original ECECAQ into Malay. The second phase, which involved 218 ECPs working in registered preschools in Kelantan, Malaysia, was conducted to validate the Malay version of ECECAQ. Five-point Likert-scaled attitude items were analysed using confirmatory factor analysis (CFA), while dichotomous-scaled knowledge items were analysed using 2-parameter logistic item response theory analysis.

**Results:**

The four-factor model for the attitude had a good fit [χ^2^ = 58.7 (48), *P* = 0.138; standardised root mean square (SRMR) = 0.044; root mean square error of approximation (RMSEA) = 0.033; comparative fit index (CFI) = 0.986; Tucker–Lewis index (TLI) = 0.981], with factor loadings (FLs) ranging from 0.414 to 0.869 and good reliability (Raykov’s rho = 0.672 to 0.878). The knowledge had a good range of difficulty and discrimination values, with acceptable reliability (Cronbach’s alpha = 0.66).

**Conclusion:**

The Malay version of ECECAQ demonstrated good validity and reliability among ECPs in Malaysia.

## Introduction

Reports of child abuse have been consistently reported in the media year after year, be it perpetrated by family members, caregivers, or relatives. These reports evoke strong emotions among the public as they witness children being battered, powerless and lifeless. The prevalence of child abuse suggests that it has become a pressing public concern. According to the World Health Organization (1), approximately 50% of children experience violence against them each year, with physical, sexual, and emotional abuse being common forms of this violence, as well as neglect. Notably, a staggering four hundred million children under the age of five regularly suffer either physical or emotional violence at home, representing around 60% of this age group (2, 3).

Cases of child abuse should not be taken lightly, as they have substantial and far-reaching effects on children’s lives, encompassing physical health, educational outcomes, interpersonal violence, and self-directed violence (1, 4, 5). The consequences of child abuse can be severe, including the risk of life-threatening injury or death. Each year, over 40,150 children under the age of eighteen years old are victims of homicide (2). However, deaths from child abuse may be under-reported as these deaths are often misclassified as deaths from other causes (2).

Cases of child abuse or suspected cases that require investigation can be reported by anyone, regardless of their relationship to the child. If a case is not supported by sufficient evidence, individuals who make reports cannot be held legally liable. In Malaysia, childcare providers, medical officers, and family members are statutorily required by law to report any suspicions of child abuse (6). The lowest reported case to the authorities was from childcare providers (7).

Despite their professional commitment to report child abuse cases, childcare providers felt demotivated to report suspected cases of child abuse, given the complexity of the problems and the reporting process. Among the reasons for non-reporting of suspected child abuse are lack of confidence regarding signs and symptoms of abuse (8, 9), limited knowledge about reporting systems, fear of social repercussions, risk of being sued by parents and others, and lack of belief in the welfare and justice system (10, 11). More recently, a study in Malaysia found that the majority of school teachers had poor knowledge, attitudes, and practices toward child abuse reporting (12), while another study also in Malaysia showed that higher knowledge is associated with the intention to report (13).

Many instruments have been developed to measure the factors associated with reporting of child abuse, but few focus specifically on childcare providers and educators. These are Early Childhood Educators Child Abuse Questionnaire (ECECAQ) (14), The Child Abuse Report Intention Scale (CARIS) (8), Teacher’s Reporting Attitude Scale (TRAS) (15), Reporting Child Abuse and Neglect Questionnaire (RCANQ) (16), and an instrument that measures perceptions and experiences related to children in need within the context of abuse (17).

Some of these instruments are unable to measure certain aspects of child abuse and its reporting. TRAS only measures the attitude towards reporting (15), while RCANQ does not measure the intention to report child abuse cases (16). The instrument by Toros and Tiirik (17) does not measure or capture the knowledge about child abuse. On the other hand, although CARIS measures knowledge, attitude, and intention to report child abuse, it was developed in the Mandarin language (8). In contrast to other measures, ECECAQ assesses the knowledge and attitude towards child abuse and its reporting, as well as the intention to report it. Developed by Dinehart and Kenny (14) in English, ECECAQ was designed for schoolteachers in kindergarten through high school settings, including those with special needs. The instrument demonstrated good internal consistency reliability, with Cronbach’s alpha values of 0.82 and 0.73 reported for the two factors within the attitude section (14). Furthermore, ECECAQ was translated into Spanish and validated by the same authors (14).

In Malaysia, limited research has been conducted on examining the knowledge, attitude, and intention toward reporting child abuse among early childcare providers (ECPs). Investigating this issue is crucial due to the low reporting rates of child abuse among ECPs despite an increasing trend of child abuse cases involving younger children. However, to the best of the researchers’ knowledge, there are currently no questionnaires available in the Malay language designed to measure the knowledge, attitude, and intention toward reporting child abuse among ECPs. Therefore, this study cross-culturally adapted ECECAQ from English into Malay and validated the questionnaire among ECPs in Malaysia. The present article details the cross-adaptation process and discusses the psychometric properties of the Malay version of ECECAQ.

## Methods

This study comprises two phases: the cross-cultural adaptation of ECECAQ into Malay and the psychometric validation of the Malay version of ECECAQ. Both phases involved ECPs working in preschools under the Kelantan Community Development Department. The designs, settings, and participants were specific for each study phase. Therefore, in this section, the original ECECAQ is described first, followed by detailed descriptions of the methods utilised for each phase of the study.

Permission for the cross-cultural adaptation of the English version of ECECAQ into the Malay language was granted by the original authors. The necessary ethical clearance was obtained from the relevant ethics committee. The study also received approval from the Director of the Kelantan Community Development Department as it involved ECPs within its jurisdiction. To ensure data confidentiality, all responses were collected anonymously, and only the research team had access to the collected data.

### Early Childhood Educators Child Abuse Questionnaire

The original English version of ECECAQ has six sections: i) socio-demographic information; ii) reporting experience; iii) education and training; iv) attitude towards child abuse and its reporting; v) knowledge of child abuse and its reporting; and vi) vignettes on child abuse (14).

The English version of ECECAQ is a self-administered questionnaire with a five-point Likert scale for 12 items in the attitude section and dichotomous responses for 19 items in the knowledge section. The attitude section comprises two domains: i) awareness of signs and ii) beliefs about reporting, both showing good internal consistency reliability (Cronbach’s alpha = 0.82 and 0.73, respectively). The Likert scale ranges from “strongly agree” (scored as 5) to “strongly disagree” (scored as 5). Negative items are reverse coded, so a higher score indicates a good attitude and vice versa. The knowledge section consists of three distinct dimensions, namely general knowledge, reporting knowledge, and legislative knowledge. Correct answers are scored as “1”, while incorrect answers are scored as “0”. The internal consistency reliability for the knowledge section was not reported in the original study (14).

### Cross-cultural Adaptation of ECECAQ into Malay

In the preparation stage, a comprehensive literature review was conducted to identify suitable instruments that could measure the objectives of this study. A search was conducted for questionnaires related to child abuse, and these were compared and evaluated. Following a thorough evaluation process, the ECECAQ questionnaire emerged as the most suitable instrument for measuring the research objectives. Furthermore, permission was obtained from the original authors to cross-culturally adapt the English version of ECECAQ into a Malay version.

**Figure 1:**
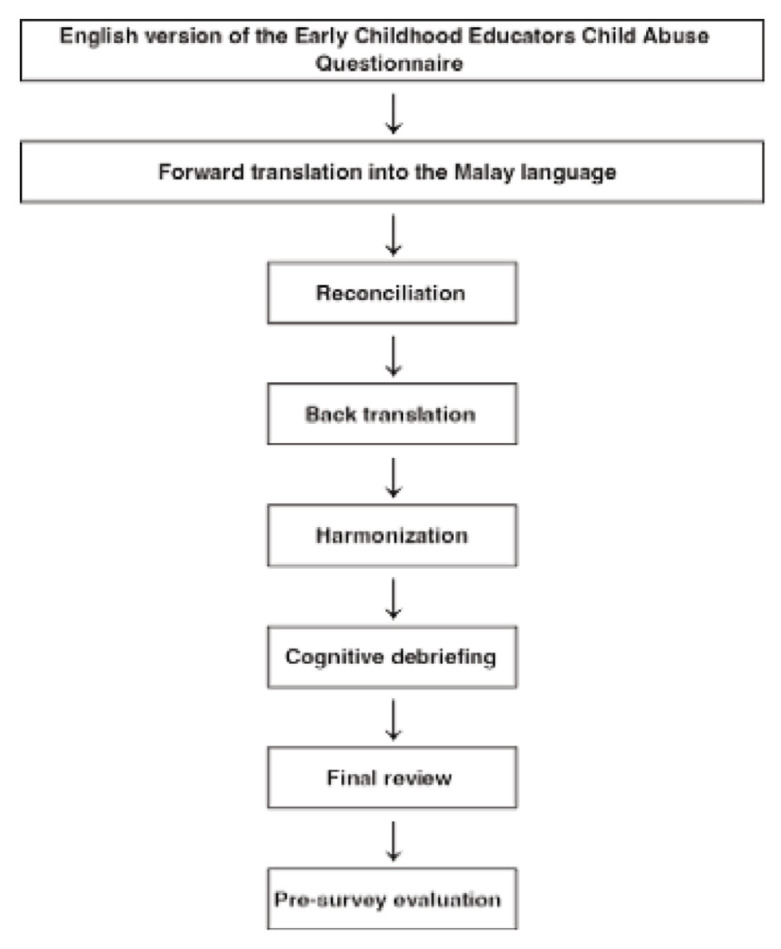
The of cross-cultural adaptation process of ECECAQ into the Malay language

This study followed the recommendations by Wild et al. (18) for translating the ECECAQ questionnaire. The translation process followed the established steps outlined in their research: forward translation, reconciliation, back translation, harmonisation, cognitive debriefing, evaluation of results from cognitive debriefing, and pre-survey evaluation (18). To prepare for this process, a meeting was convened to discuss each item in the original English version, resulting in a preliminary adapted version that took into account relevant Malaysian cultural aspects. Items relevant to Malaysian culture and context were preserved, while those that were less relevant were modified or removed. The meeting involved an expert panel consisting of an expert with extensive experience in questionnaire development, familiar with all stages of translation and validation processes; a public health specialist specialising in children and adolescents, providing insights from a clinical perspective; a representative from the state’s Department of Social Welfare, offering expertise on legal and policy aspects related to child abuse; and a representative from the state’s Community Development Department, sharing knowledge about existing practices for preventing child abuse in preschool settings. The members of this meeting formed an expert committee, which was later involved in the subsequent process. A summary of the cross-cultural adaptation process is shown in Figure 1.

#### Forward Translation

The preliminary, adapted version of the original English questionnaire was distributed to two independent translators, who were tasked with translating it into Malay. The translators were an officer from the Department of Social Welfare and a linguistic teacher in the literacy unit at Universiti Sains Malaysia, both of whom were fluent in both languages. Both translators were blinded to the purpose of the study, ensuring its neutrality. Furthermore, they were briefed on the conceptual basis of the questionnaire and required to utilise common Malay phrases and terms for translation. The two translators worked independently on their respective tasks, and each was given one month to produce a Malay translation of the questionnaire.

#### Reconciliation

The Malay versions underwent review during the reconciliation stage by the expert committee. However, neither translator attended the meeting due to work constraints. The discrepancies identified between each version of the Malay translations were subsequently discussed, and a reconciled version was reached.

#### Back Translation

The reconciled version was then back-translated by two independent translators, one a postgraduate student pursuing a Doctor of Public Health at Universiti Sains Malaysia and the other an English teacher at a primary school in Malaysia. Both were bilingual and worked independently on their tasks. Additionally, neither translator had access to the concept or purpose of the study, nor did they know the original English version of ECECAQ. They were given one month to complete their translations.

#### Harmonisation

The two forward translations in Malay and their corresponding back translations in English were reviewed together and discussed. This stage is crucial for identifying any loss of meaning during translation and ensuring that the content remains contextually equivalent to the original English version. Additionally, during this meeting, because one translator was unable to attend due to work commitments, the other translator was not invited to join the meeting to prevent potential bias.

The same expert committee participated in this stage, with the exception of the state’s Department of Social Welfare representative. Two external experts were invited to provide unbiased perspectives: a postgraduate student completing his Doctor of Public Health at Universiti Sains Malaysia, and a representative from the literacy unit of the university. Due to the complexity of the matter, two meeting sessions were held. At the end of the meeting, a harmonised Malay-translated version of ECECAQ was produced for testing in the next stage.

#### Cognitive Debriefing

Five ECPs were selected for the cognitive debriefing stage. All respondents were women aged between 28 and 60 years old, with diverse educational backgrounds and work experiences. Each participant was given the Malay version of the ECECAQ questionnaire to complete independently. Subsequently, face-to-face interviews were conducted to examine their thought processes during the questionnaire completion. The interview sessions followed a structured list of questions based on guidelines provided by McDonald et al. (19), ensuring that all important points and concerns were addressed systematically. This approach facilitated a structured and consistent cognitive debriefing process.

Interview sessions were recorded to assist the researcher in the analysis. At this stage, the comprehensibility and cognitive equivalence of the questionnaire were assessed, and any inappropriate items or other issues that arose with the questionnaire were discussed. Respondents were also given alternative phrases or words for certain questions. Following their feedback, more suitable phrases were then chosen. Additionally, the time it took to complete the questionnaire was also tested.

#### Final Review

Feedback and findings from the cognitive debriefing process were discussed in the expert committee. The results were compared to the original English version, highlighting inconsistencies that were then addressed to improve the accuracy of the Malay translation and better reflect the original content.

#### Pre-survey Evaluation

Lastly, the pre-final ECECAQ was tested on 30 selected ECPs in Kota Bharu district. All respondents were women between the ages of 24 and 59 years old, with diverse educational backgrounds and work experiences. They gathered at a Child Development Centre’s facility in Kota Bharu, where they were briefed about the study’s purpose and the questionnaire’s use. Before data collection commenced, written informed consent was obtained from each participant. Respondents then received the pre-final Malay version of the ECECAQ questionnaire and a feedback form. They completed the questionnaire individually, and each respondent’s completion time was recorded.

The feedback and comments provided by respondents were discussed with the expert committee, who compared them to the original English version of the ECECAQ. Based on the comparison, necessary revisions were made to produce a final version of the Malay translation of ECECAQ that was designed to be tested on 200 participants in the subsequent validation study.

### Validation of the Malay Version of ECECAQ

#### Study Setting and Participants

This cross-sectional study involved ECPs working in preschools located in Kota Bharu and Tumpat under the Kelantan Community Development Department. According to guidelines by Kline (20) and Edelen and Reeve (21), a minimum of 200 participants is required for both confirmatory factor analysis (CFA) and two-parameter logistic item response theory (2PL-IRT) analysis. Since the number of available ECPs was approximately equal to this requirement, no specific sampling method was employed.

A total of 238 ECPs were identified as potential participants. All those who understood Malay were invited to take part in the study. Data collection took place at the Community Development Department facilities in the selected districts, where identified ECPs were invited for data collection sessions. For individuals unable to attend these sessions, they were approached individually at their workplaces. Each participant was informed about the purpose of the research and provided written consent before participating.

#### Statistical Analyses

Data were analysed using the R statistical programming language version 4.0.2 (22). Two sections in ECECAQ, namely the attitude and knowledge sections, required further statistical analyses to provide internal structure validation for these sections. The remaining sections were deemed suitable for qualitative assessment alone, as thoroughly conducted during the preceding cross-cultural adaptation process. The attitude section was analysed using CFA, while the knowledge section was analysed using the 2PL-IRT analysis.

The CFA was conducted using the *lavaan* R package (23). A robust maximum likelihood estimation method was used because the data were not normally distributed. Model fit was evaluated based on five fitness indices shown in Table 1. Localised areas of misfit were identified by standardised residuals (SR) and modification indices (MI). An SR value greater than |2.58| and an MI value greater than 3.84 indicated misfits (24). A factor loading (FL) value greater than 0.3 was considered acceptable (25). Factor-to-factor correlation values less than 0.85 were used to indicate distinct factors (24). For internal consistency reliability, Raykov’s rho value of ≥ 0.7 was accepted (27).

The 2PL-IRT analysis was carried out using the *ltm* R package (28) to estimate the difficulty and discrimination for each item. Difficulty refers to the probability that a respondent will correctly answer an item, with acceptable difficulty values ranging from −3 to +3 (29). Discrimination refers to an item’s ability to differentiate between weak and strong respondents (29). The range of discrimination values in this study is as follows: very low discrimination (0.01 to 0.34), low discrimination (0.35 to 0.64), moderate discrimination (0.65 to 1.34), high discrimination (1.35 to 1.69), and very high discrimination (≥ 1.7) (30). Item fit was determined by a chi-square goodness-of-fit test for each item (31). Unidimensionality was determined using modified parallel analysis (32). For internal consistency reliability, a Cronbach’s alpha value of ≥ 0.65 was accepted (33).

## Results

### Cross-cultural Adaptation Process of ECECAQ

During the translation process, certain words and phrases were modified to better fit the context. Additionally, specific items, responses, and scenarios were adapted to align with the Malaysian setting. In summary, all items in the attitude section were retained because they were considered important and relevant by the expert committee. Two questions from the knowledge section were removed: one related to statistics and another concerning regular reporters. The format and layout of the original questionnaire remained unchanged, mirroring the structure of the English version as closely as possible.

#### Forward Translation

Neither translator reported any significant difficulties in translating sentences or phrases. Both translators preserved the format and layout of the original questionnaire.

#### Reconciliation

No major issues were identified during this step. Content-wise, both translated versions remained similar to the original and relevant to Malaysian culture. In terms of meaning, both versions conveyed the same message in English and Malay.

However, a few items were revised and rephrased by the committee to make statements or questions simpler and more comprehensible. For example, the expert committee agreed that “corporal punishment” should be translated into *hukuman fizikal* rather than *hukuman rotan* or *hukuman dera*. This translation was chosen because the words *rotan* (caning) or *dera* (abuse) have negative connotations in the Malay language, as they may imply a severe form of punishment or abuse.

#### Back Translation and Harmonisation

During the harmonisation process, many words, phrases, and sentences that were not equivalent to the original English version were identified and corrected. The committee agreed to drop two questions from the knowledge section. The first question dropped was Question 8, which asked about statistics of child abuse victims per year. The original question referred specifically to America’s context and did not fit the Malaysian setting due to differences in available data. The second question removed was Question 12, which asked about the main reporter of child abuse cases. The committee agreed that respondents would not be able to answer this question accurately because of limited information shared by the local media. Additionally, these questions were deemed unnecessary for achieving the study’s objectives.

#### Cognitive Debriefing, Final Review and Pre-survey Evaluation

Based on feedback from participants, several wordings and formats of the Malay version of ECECAQ were edited. For example, some respondents suggested adding an “others” option in the response section for vignettes to allow them to provide additional actions or reasons that were not covered by existing options.

### Validation of the Malay Version of ECECAQ

A total of 238 questionnaires were distributed to all ECPs working in Community Development Department facilities in Kota Bharu and Tumpat. A total of 230 questionnaires were returned. Twelve questionnaires were excluded from the analysis due to missing data, leaving a final dataset of 218 questionnaires, which represents a response rate of 91.6%.

The majority of ECPs in this study were female (99.1%) and Malay (99.1%), with a mean age of 40.9 years (SD = 11.2). Most respondents (77.5%) had completed tertiary-level education. More than one-third of the participants (37.6%) had worked in the field for over 20 years. The vast majority (99.1%) were responsible for toddlers aged two to four years. Only one respondent (0.5%) ever reported a case of child abuse to the authorities. Just 21.6% of respondents had attended training on child abuse. Table 2 provides further details on the socio-demographic characteristics of the respondents.

#### Confirmatory Factor Analysis

The CFA was performed according to the two-factor model outlined in the original ECECAQ. It was found that none of the fit indices met the recommended cut-off values, suggesting that the initial model did not adequately fit the data (Table 3). Consequently, the model was redefined as a four-factor structure for the Malay version of ECECAQ. The new factors are Perception (items A5, A8, and A12), Symptoms (items A9, A10, and A11), Support System (items A3 and A4), and Responsibility (items A2, A6, and A7). This revised model demonstrated a good fit according to all the fit indices provided (Table 3). The FLs for these items were acceptable, ranging from 0.414 to 0.869.

Table 4 shows the FLs and reliability values for the new four-factor model. There were seven suggested changes in model specification based on MI values > 3.84, involving items A1, A2, A3, A4, and A11. Additionally, no SR exceeded |2.58|. After reviewing the content of the items with the suggested changes and considering that all SR were below the cut-off value, no modifications were made to the four-factor model. The construct reliability for all factors was good, as indicated by Raykov’s rho values: 0.672 (Perception), 0.878 (Symptoms), 0.820 (Support System), and 0.672 (Responsibility). The factor-to-factor correlations were all less than 0.85, indicating that the factors are distinct from each other. The correlations between each pair of factors ranged from 0.49 to 0.80.

#### Item Response Theory

In the 2PL-IRT analysis, all items fell within the difficulty range of −3 to +3, except for item K14, which had a difficulty value of 7.53. The discrimination values for the items ranged from very low (K14 = 0.23) to very high (K12 = 3.23) (Table 5). Items with very high discrimination ability were K1, K3, and K12. Items with moderate discrimination ability included K4, K5b, K8, K9, K11c, K15, and K16. Items with low discrimination ability were K2, K5c, K5d, K6, K7, K10, K13, and K17. Meanwhile, items with very low discrimination ability were K5a, K11b, and K14.

At an ability range of −3 to +3, the test generated information of 80.5%. For individuals with an ability level of +4, the expected true score was approximately 19. Conversely, for those with an ability level of −4, it is expected that they would get at least three items correct.

Based on the chi-square goodness-of-fit test, four items did not fit the model (*P* < 0.05): K2, K4, K5c, and K8. However, these items were retained because their difficulty was within range, their discrimination ranged from low to moderate, and their content was deemed important. For the modified parallel analysis, the *P*-value was ≥ 0.05, indicating that the model was unidimensional. The internal consistency reliability value was acceptable (Cronbach’s alpha = 0.66).

## Discussion

ECECAQ was developed to measure knowledge and attitude towards child abuse and its reporting, as well as the intention to report child abuse among childcare providers. Since ECECAQ was not available in Malay, this study involved translating, adapting, and validating it for use with the Malaysian population. To the best of the researchers’ knowledge, this is the first study to adapt and validate ECECAQ in Malaysia. Based on the presented results, the Malay version of ECECAQ has been found to be valid and reliable when tested among ECPs in registered preschools in Kelantan.

### Attitude Section

The original version of ECECAQ used exploratory factor analysis (EFA) to determine the internal structure of the attitude section. However, the Malay version utilised CFA to verify the two-factor model specified in the original version. CFA provided stronger support for validating the factor structure (24). Nevertheless, the original two-factor model did not fit the data. Although there is no specific guidance on the number of fit indices that must be met for a model to be deemed fit (34), a model that fails to meet the criteria of any suggested indices indicates that it does not fit well. For this reason, the original two-factor model was respecified as a four-factor model. The revised model demonstrated good fit and met the criteria for model fit based on all four suggested fit indices.

The FL values for the items across all factors ranged from 0.414 to 0.869. Although the original authors did not report FL values, the FL values in this study were acceptable, meeting the recommended cut-off value of > 0.3 (27). The internal consistency reliability, assessed using Raykov’s rho, was also good for all four factors, with values ranging from 0.672 to 0.878. Raykov’s rho tests construct reliability by considering the correlated error covariances (35).

The four-factor model in the attitude section of the Malay version of ECECAQ is: i) Perception, measuring ECPs’ interpretation of issues surrounding child abuse; ii) Symptoms, measuring ECPs’ awareness of signs and symptoms of child abuse; iii) Support System, measuring ECPs’ perceived assistance to report child abuse; and iv) Responsibility, measuring ECPs’ awareness of the graveness of the child abuse issue and their commitment to lodge a report.

### Knowledge Section

Initially, there were 19 items in the knowledge section of ECECAQ. However, following a discussion with the expert committee, two items were removed: questions on child abuse statistics and common reporters. The committee decided that these items were too specific and likely to yield incorrect answers, potentially affecting the scale’s validity.

The authors of the English version of ECECAQ did not measure internal consistency for the knowledge section due to inconsistent correct answers across each dimension (14). For the Malay version of ECECAQ, a 2PL-IRT analysis was conducted on 17 items in the knowledge section. Generally, all items had good difficulty and discrimination values. Although some items exhibited low to very low discrimination values, they were retained because their difficulty levels were within acceptable ranges and the content was deemed important. Consequently, no further items were removed from the Malay version of ECECAQ after the 2PL-IRT analysis.

### Limitations

There were several limitations to this study. First, it was conducted among ECPs from only one agency, which may not adequately represent all ECPs in Malaysia. Future research should cross-validate the Malay version of the ECECAQ among ECPs from other agencies and the private sector. Second, 99.1% of respondents were of Malay ethnicity, limiting the validity of the instrument specifically to Malays. To address this limitation, future studies should include ECPs with diverse ethnic backgrounds. Third, 99.1% of the respondents were female. However, since ECPs are predominantly female, as observed in the original ECECAQ study (14), with 95% being female, this might not affect the generalisability of the Malay version of ECECAQ among its target population. Fourth, certain phrases or words in English may not have been accurately translated into Malay, leading to inconsistencies in meaning and semantic equivalence issues. The research team appointed a committee of experts to oversee the translation process to minimise these errors, but further refinement might be necessary. By addressing these limitations, future studies can enhance the generalisability and reliability of the ECECAQ instrument across different populations and settings.

## Conclusion

This study provides evidence of the validity and reliability of the Malay version of ECECAQ. The results indicate that the self-administered instrument is valid and reliable for assessing knowledge and attitude towards child abuse and its reporting, as well as the intention to report child abuse among childcare providers in Malaysia. However, given the current limitations in the population and setting studied, cross-validation studies of the Malay version of ECECAQ in different populations and settings are recommended.

## Figures and Tables

**Table 1:** Cut-off values of fit indices for the CFA

Category	Fit index^a^	Cut-off^b^
Absolute fit	χ^2^	*P* > 0.05
	SRMR	≤ 0.08

Parsimony correction	RMSEA and its	≤ 0.08,
	90% CI	CFit *P* > 0.05

Comparative fit	CFI	≥ 0.95
	TLI	≥ 0.95

Notes:

a Fit indices recommended by Kline (20) and Brown (24);

b Cut-off values recommended by Brown (24) and Schreiber et al. (26);

SRMR = standardised root mean square; RMSEA = root mean square error of approximation; CFI = comparative fit index; CFit = close fit; CI = confidence interval; TLI = Tucker–Lewis index

**Table 2:** Socio-demographic characteristics of respondents (*n* = 218)

Variable	Mean (SD)	*n* (%)
Age (years)	40.9 (11.2)	

Gender		
Male		2 (0.9)
Female		216 (99.1)

Race		
Malay		216 (99.1)
Siam		2 (0.9)

First Language		
Malay		215 (98.6)
Siamese		3 (1.4)

Education level		
Up to secondary level		49 (22.5)
Tertiary level		169 (77.5)

Currently further study		
No		201 (92.2)
Yes		17 (7.8)

Working period		
Less than or equal to 20 years		136 (62.4)
More than 20 years		82 (37.6)

Children under care (*n* = 218)		
Equal or less than 12 months old		1 (0.5)
12 to 24 months old		2 (0.9)
2 to 4 years old		216 (99.1)
5 to 6 years old		24 (11.0)

Ever reported case to authority		
No		217 (99.5)
Yes		1 (0.5)

Ever had training on child abuse		
No		171 (78.4)
Yes		47 (21.6)

**Table 3:** Comparison of fit indices between the original and modified CFA models

Model	χ^2^ (df)	*P*	SRMR	RMSEA	90% CI	CFI	TLI
Original two factors	187.5 (53)	< 0.001	0.081	0.108	0.091, 0.125	0.830	0.789
New four factors	58.7 (48)	0.138	0.044	0.033	0.000, 0.058	0.986	0.981

Notes: SRMR = standardised root mean square; RMSEA = root mean square error of approximation; CFI = comparative fit index; TLI = Tucker–Lewis index

**Table 4:** FL and reliability values of the new four-factor CFA model

Factor	Item	FL	Raykov’s rho
Perception	A1 Parental rights in disciplining a child	0.444	0.672
	A5 Child abuse is a serious problem in the community	0.515	
	A8 Family can sue any unfounded report	0.618	
	A12 EPCs should be allowed to use corporal punishment	0.555	

Symptoms	A9 Aware of the signs of child neglect	0.600	0.878
	A10 Aware of the signs of physical abuse	0.414	
	A11 Aware of the signs of sexual abuse	0.636	

Support system	A3 Aware of school’s reporting procedure	0.422	0.820
	A4 Director would support to file a report	0.573	

Responsibility	A2 ECPs should be mandated to report abuse	0.802	0.672
	A6 Child abuse is a serious problem in my school	0.842	
	A7 ECPs should have an obligation to report abuse	0.869	

**Table 5:** FL and reliability values of the new four-factor CFA model

Item	Difficulty	Discrimination	χ^2^ (*df =* 8)	*P-*value
K1 Calling the abuse hotline to make a report of suspected abuse can be done	−1.52	2.12	7.10	0.526

K2 Complication of failing to make a report of abuse as a mandated reporter	−1.92	0.50	20.91	0.007

K3 Types of abuse when parent fails to provide adequate shelter and clothing for a child	−1.10	2.46	12.29	0.139

K4 Types of abuse when a non-accidental injury resulted from acts by the parents	−1.30	0.67	20.33	0.009

K5 Medium to report child abuse				
K5a By e-mail	−1.93	0.34	12.18	0.144
K5b By phone	−1.72	1.03	6.23	0.622
K5c By mail	−0.53	0.63	15.66	0.048
K5d All of the above	−0.30	0.63	11.91	0.155

K6 Make a report of abuse in good faith and it proves unfounded	0.08	0.39	8.84	0.356

K7 The most commonly identified perpetrators	1.13	0.53	7.69	0.464

K8 Mandated reporters of abuse in Malaysia according to law	1.00	0.66	22.78	0.004

K9 Children’s peculiarity in disclosing abuse	−1.25	0.71	11.83	0.159

K10 The biggest indicators of sexually abused child	0.51	0.62	6.47	0.594

K11 Children may recant their disclosure of abuse due to				
K11b Pressure from family members	−1.27	0.28	9.77	0.281
K11c Fear of consequences	−2.01	0.67	7.45	0.489

K12 Identity of the person who reports child abuse is protected under the law	−1.39	3.23	8.28	0.407

K13 In case of a recurrent abuse case that have been reported previously, what will be the best action	−2.31	0.60	7.00	0.537

K14 A must have criterion to file a report	7.53	0.23	8.68	0.370

K15 Types of law infringement in case of a failure to report child abuse	−0.97	0.65	9.16	0.329

K16 Types of abuse involved terrorising, shaming, and degrading a child repeatedly	−1.54	0.74	7.10	0.525

K17 Information needed to file a report	−2.02	0.62	4.56	0.803

## Data Availability

Since ECECAQ comprises many detailed items across multiple sections, the cross-culturally adapted items and statements contained in the Malay version of ECECAQ cannot be included in this article. A formatted copy of the Malay version of ECECAQ can be requested from any of the authors via their respective emails. An earlier draft of this manuscript is available in the form of chapter in the doctorate thesis of one of the authors, accessible at http://eprints.usm.my/49581/.
